# Dynamics of depressive states among university students in Japan during the COVID-19 pandemic: an interrupted time series analysis

**DOI:** 10.1186/s12991-023-00468-9

**Published:** 2023-10-10

**Authors:** N. Shiraishi, M. Sakata, R. Toyomoto, K. Yoshida, Y. Luo, Y. Nakagami, A. Tajika, T. Watanabe, E. Sahker, T. Uwatoko, T. Shimamoto, T. Iwami, T. A. Furukawa

**Affiliations:** 1 Department of Psychiatry and Cognitive-Behavioral Medicine, Nagoya City University Graduate School of Medical Science, 1 Kawasumi, Mizuho-Cho, Mizuho-Ku, Nagoya, 467-8601 Japan; 2 Department of Health Promotion and Human Behavior, Graduate School of Medicine/School of Public Health, Kyoto University, Kyoto, Japan; 3 Kyoto University Health Service, Kyoto, Japan; 4 Medical Education Center, Kyoto University Graduate School of Medicine, Kyoto, Japan; 5 Department of Neuropsychiatry, Kyoto University Hospital, Kyoto, Japan

**Keywords:** Depression, *Gogatsubyou*, Higher education, Novel coronavirus, Student apathy

## Abstract

**Background:**

The coronavirus disease 2019 (COVID-19) pandemic was reported to have increased depression among university students which was associated with impairments in their campus lives. This study examined changes in depressive states among Japanese university students during the COVID-19 pandemic.

**Methods:**

A secondary data analysis from a factorial randomized controlled trial involving smartphone-based cognitive-behavioral therapy was performed. Six cohorts (*N* = 1626) underwent an 8-week intervention during the spring or autumn of 2019–2021, with a 9-month follow-up. We evaluated participants’ depressive states weekly using the Patient Health Questionnaire-9 (PHQ-9) during the intervention, with monthly evaluations thereafter. The follow-up periods included Japan’s four states of emergency (SOEs) to control COVID-19. Hypothesizing that SOEs caused a sudden worsening of depressive states, Study 1 compared the cohorts’ PHQ-9 scores, and Study 2 employed time series analysis with a mixed-effects model to estimate identified changes in PHQ-9 scores.

**Results:**

Although no changes in depressive states were observed in relation to the SOEs, Study 1 identified sudden increases in PHQ-9 scores at the 28-week evaluation point, which corresponded to the beginning of the new academic year for the three autumn cohorts. In contrast, the three spring cohorts did not exhibit similar changes. Study 2 showed that, for all three autumn cohorts (*n* = 522), the 0.60-point change was significant (95% CI 0.42–0.78; *p* < .001) at 28 weeks; that is, when their timeline was interrupted.

**Conclusions:**

While the results do not indicate any notable impact of the SOEs, they highlight the influence of the new academic year on university students’ mental health during COVID-19.

*Trial registration* UMIN, CTR-000031307. Registered on February 14, 2018.

**Supplementary Information:**

The online version contains supplementary material available at 10.1186/s12991-023-00468-9.

## Background

Depression is a significant health issue, impacting young and older populations. Previous studies [1, 2] showed that it was particularly prevalent in university students. University students experience more depressive states compared to the general population. The prevalence of depression among university students worldwide was estimated as 32% [3], while community prevalence was approximately 13% [4]. Specific factors associated with depression in university students include age and sex [5]; academic level [6]; social support [7]; financial difficulties [8]; lifestyle-related behaviors such as exercise, breakfast, drinking, and smoking [9, 10]; history of mental illness [11]; and stressful events [12, 13]. Moreover, World Mental Health surveys have shown that university students with depression tend to perceive distress across multiple life areas, including their relationships, home and school, and their financial situations [14]. This supports the evidence that students with depression exhibit an increased risk of severe impairment in housework, schoolwork, and close/social relationships [15].

Depression-related impairments among students have likely intensified since early 2020 when the coronavirus (COVID-19) pandemic imposed additional stressors. In this ongoing context, it is important to understand how COVID-19 affects depression in university students while also considering associated factors. Various studies have identified rates of depression in student samples during the pandemic; notably, congruent rates of 34%, 34%, 39%, and 37% were reported by Chang et al. [16], Deng et al. [17], Li et al. [18], and C. Wang et al. [19], respectively. These rates are comparatively higher than pre-pandemic rates [1].

Concurrently, acknowledging that COVID-19’s impacts may differ across countries is important. Two reviews found that the prevalence of depressive symptoms in Chinese students was approximately 2.4 times lower than in non-Chinese students [18, 19]. This substantial difference highlights the importance of determining depression prevalence in a specific country or region. Indeed, studies have also observed variations in depression severity during COVID-19 between students in different Asian countries, including China, South Korea, and Japan [20]. Notably, the Japanese government implemented four states of emergency (SOEs) to control COVID-19 between April 2020 and September 2021. The four SOEs were initiated by the Japanese government and allowed it to take swift measures to protect the public from the spread of COVID-19. Each SOE declaration during this period was in response to surging COVID-19 cases, with the government monitoring the situation and determining when to lift or reimplement restrictions based on infection rates. Japanese universities rarely conducted in-person education on campus during those periods. The alternative online education may have had consequences that influenced feelings of depression owing to the induction of a more isolated and sedentary lifestyle [21]. These effects are reflected in two university surveys that showed that students manifested more depressive symptoms in 2020 compared to previous years [22, 23]. However, this influence was not seen everywhere, as another university survey in Japan [24] showed reduced depressive symptoms among students in 2020 compared to 2019. The existing evidence does not fully clarify how COVID-19 impacted the depressive states experienced by university students in Japan.

In this study, we conducted a secondary analysis of data from the Healthy Campus Trial (HCT), a full-factorial randomized controlled trial that employed smartphone-based cognitive-behavioral therapy (CBT) to help university students improve their mental health [25, 26]. Details are available in the protocol study by Uwatoko et al. [27]. The HCT began in the autumn of 2018 and continued during the COVID-19 pandemic. A total of 1626 students were randomly allocated to the presence or absence of each of the five CBT elements: self-monitoring, cognitive restructuring, behavioral activation, assertive communication, and problem-solving. Depressive states were evaluated weekly during the 8-week intervention period, then every 4 weeks during the 44-week follow-up period. The distinct efficacy of the five elements and their interactions were estimated using a mixed-effects repeated-measures analysis. Based on the estimation, Sakata et al. [25] examined the most beneficial combinations of these elements. The overall aim of this study was to investigate the dynamics of depressive states in university students both before and through the four SOEs to control COVID-19 during the follow-up period.

## Methods

This study consisted of two components (Study 1 and Study 2), which are outlined below. Written informed consent was obtained from students who participated in the HCT. We used data from the trial, with the approval of the Ethics Committees of Kyoto University Graduate School of Medicine (no. C1357) and Nagoya City University Graduate School of Medical Sciences (no. 46-19-0006).

### Study 1 aims

Study 1 explored how the four SOEs impacted depressive states during the HCT follow-up period. We hypothesized that each SOE exacerbated depressive states following their declaration, with the first SOE having the largest effect (level change).

### Study 1 methods

#### Participants

In the HCT, students from two public and three private universities were recruited in spring (April to May) or autumn (the latter half of September to the first half of November). The inclusion criteria were (1) ages 18–39 years; (2) enrollment in an undergraduate or graduate course; and (3) possession of a personal smartphone. The exclusion criteria were (1) Patient Health Questionnaire-9 (PHQ-9) scores of ≥ 15 or 10–14 with suicidal ideation for more than half of the days, and (2) currently receiving professional treatment for mental health problems. Students were recruited on campus with promotional materials such as flyers, pamphlets, and giveaways such as pocket tissues. Social media and websites were also used for recruitment. From September 2018 through May 2021, the HCT enrolled students with and without subthreshold depression. As the Japanese academic year begins on April 1, newly enrolled students were excluded in the spring; first-year students typically need several months to adjust to campus life.

#### Exposure

The HCT recruitment produced three autumn cohorts and three spring cohorts. All six cohorts were engaged in the intervention period (0 [baseline] to 8 weeks) and the follow-up period (8–52 weeks). Participants were exposed to the four SOEs during follow-ups. The first SOE (between April 7 and May 25, 2020) occurred during the follow-up periods for the spring and autumn cohorts from 2019. After the intervention period, the spring cohort in 2020 overlapped with the second SOE (between January 8 and March 21, 2021) and the third SOE (between April 25 and June 20, 2021). The 2020 autumn cohort included the second, third, and fourth SOEs (between July 12 and September 30, 2021). The fourth SOE occurred during the 2021 spring cohort’s follow-up period. Contrastingly, no SOEs existed during the 2018 autumn cohort, for which the follow-up period had been completed prior to COVID-19.

The PHQ-9 was used to examine the effects of all four SOEs on depressive states. The PHQ-9 items correspond to the criteria for a major depressive episode according to the Diagnostic and Statistical Manual of Mental Disorders (4th ed.; DSM-IV) [28]. In the HCT follow-up period, participants self-rated each item on a 4-point rating scale (ranging from 0 = *not at all* to 3 = *nearly every day*) every four weeks. Depressive-state severity was thus indicated by total scores of 0–4 (*no symptoms*), 5–9 (*mild*), 10–14 (*moderate*), 15–19 (*moderately severe*), and 19–27 (*severe*) [29]. Muramatsu et al.'s study [30] confirmed the validity and reliability of the Japanese version of the PHQ-9.

#### Data analysis

For all six cohorts, we calculated the representative values of PHQ-9 total scores at every 4-week evaluation point. We then plotted the mean values to identify changes in the trajectories of their depressive states. In this context, we compared the autumn cohorts in 2019 and 2020 to the autumn cohort in 2018, which allowed us to explore how the two trajectories changed based on the first SOE and other SOEs, respectively. Following these comparisons, we validated the effects of the four SOEs according to similar trajectory changes in the spring cohort in 2019 for the first SOE and spring cohorts in 2020 and 2021 for other SOEs.

### Study 2 aims

Based on the results of Study 1 (described below), Study 2 examined how the beginning of a new academic year impacted depressive states. Here, we hypothesized that depressive states had suddenly exacerbated at the beginning of April, with the effects lasting for a few months.

### Study 2 methods

#### Participants and exposure

We grouped 1,626 HCT participants into autumn and spring cohorts. To compare the distribution of categorical variables between the two seasonal cohorts, a Chi-squared (χ^2^) test was employed. For the autumn cohorts, we set a 28-week evaluation point to examine the impacts of the environment in which the timeline was interrupted by the beginning of a new academic year. For the spring cohorts, we did not evaluate points at the beginning of April (after the intervention periods).

#### Data analysis

We conducted a segmented regression analysis using (1) interrupted time series (ITS) data from the autumn cohorts and (2) non-interrupted comparative data from the spring cohorts during the follow-up periods. We built two mixed-effects linear regression models to estimate average exposure effects and simple linear effects on the trajectory of PHQ-9 scores for the autumn and spring cohorts, respectively. The former model included variables representing time, level change, and slope change (the interaction between time and level change). We also included random effects for student-specific intercept and slope into the model with a better fit, based on a log likelihood-ratio test, versus the intercept-only model. We used R version 4.2.1 (2022-06-23) to perform all analyses, with a two-sided significance level of 5%.

## Results

### Study 1 results

**Fig. 1:**
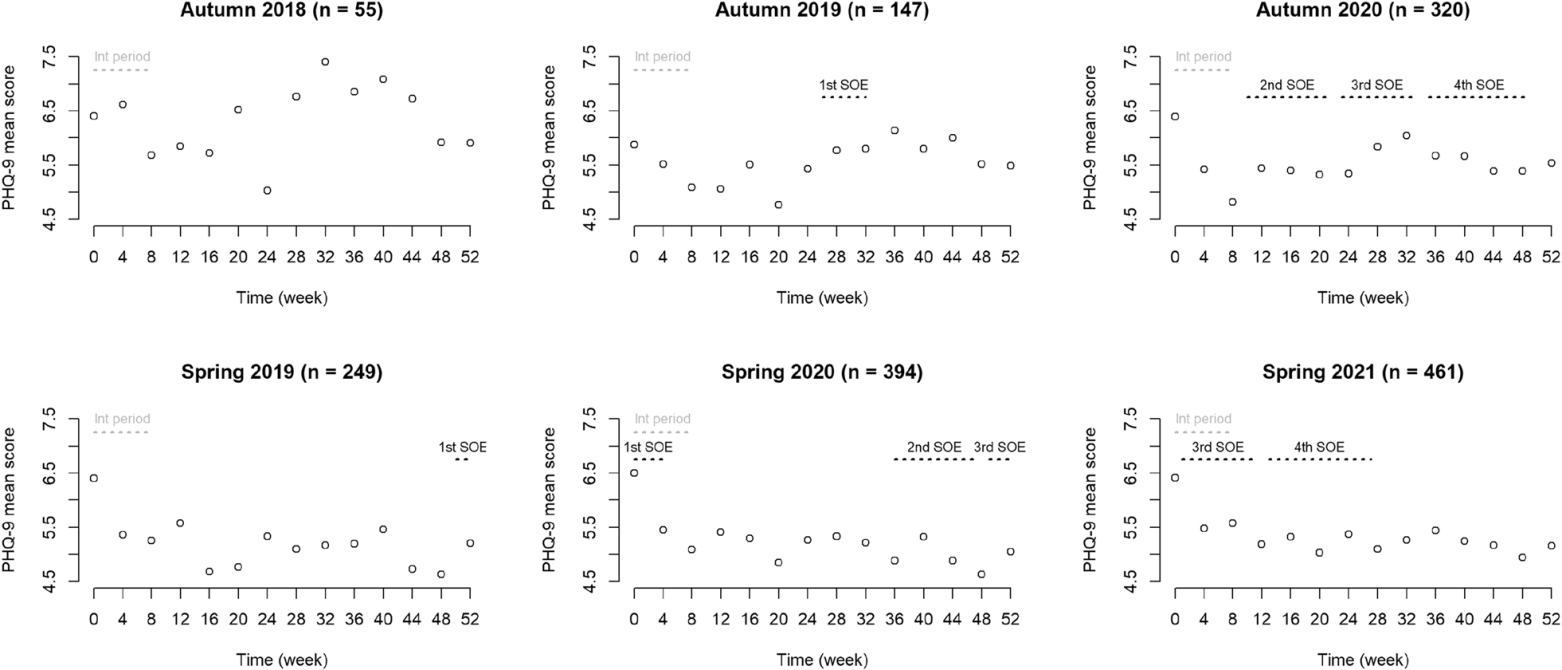
Trajectories of depressive states among university students in the HCT’s individual cohorts (2018–2021). *HCT* Healthy Campus Trial, *Int period* intervention period, *PHQ-9* Patient Health Questionnaire-9, *SOE* state of emergency

Table 1 lists the sociodemographic characteristics of each HCT cohort; the total sample included 1,626 students (mean age = 21.5 years; sex ratio = 0.74). Notably, a range of characteristics varied between cohorts; there were differences in sex ratios, academic levels, living arrangements, part-time employment, and drinking habits (Additional file 1). For example, sex ratios exceeded one in the 2018 and 2019 autumn cohorts and the 2019 spring cohorts. Nevertheless, the mean values of PHQ-9 scores exhibited two trajectory patterns during the follow-up periods across the six cohorts (Fig. 1 and Additional file 2). For the 2019 autumn cohort, the first SOE overlapped with an upward shift in PHQ-9 scores. The 2020 autumn cohort included the second through fourth SOEs; however, their PHQ-9 scores only shifted upward in conjunction with the third SOE. The trajectory of all the autumn cohorts appeared to be interrupted at the 28-week evaluation point, which coincided with the beginning of a new academic year in April. Furthermore, we observed no level change in PHQ-9 scores during the follow-up period for the spring cohort in 2019 for the first SOE, nor for the spring cohorts in 2020 and 2021 for other SOEs. The trajectories of all spring cohorts indicated a downward trend following the intervention periods.

**Table 1:** Characteristics of university students in the autumn and spring cohorts of the Healthy Campus Trial

Characteristics	Total *n* = 1626	Autumn *n* = 522	Spring *n* = 1104	χ^2^ (*p*-value)
Age, mean (range), years	21.5 (18–39)	21.4 (18–35)	21.6 (18–39)	
Sex, *n* (%)				
Male	693 (42.6)	217 (41.6)	476 (43.1)	0.29 (0.59)
Female	933 (57.4)	305 (58.4)	628 (56.9)	
Academic level, *n* (%)				
Undergraduate	1,250 (76.9)	416 (79.7)	834 (75.5)	4.53 (0.10)
Master	291 (17.9)	86 (16.5)	205 (18.6)	
Doctorate	85 (5.2)	20 (3.8)	65 (5.9)	
Living status, *n* (%)				
Living with family	624 (38.4)	204 (39.1)	420 (38.0)	2.66 (0.45)
Living alone	902 (55.5)	284 (54.4)	618 (56.0)	
Living in a dormitory	70 (4.3)	27 (5.2)	43 (3.9)	
Others	30 (1.8)	7 (1.3)	23 (2.1)	
Part-time employment, *n* (%)				
None	365 (22.4)	110 (21.1)	255 (23.1)	2.30 (0.68)
A few times/year	148 (9.1)	54 (10.3)	94 (8.5)	
One to three times/month	330 (20.3)	102 (19.5)	228 (20.7)	
Two to three times/week	656 (40.3)	214 (41.0)	442 (40.0)	
More than four times/week	127 (7.8)	42 (8.0)	85 (7.7)	
Breakfast, *n* (%)				
Rarely	171 (10.5)	45 (8.6)	126 (11.4)	5.32 (0.07)
Sometimes	581 (35.7)	177 (33.9)	404 (36.6)	
Daily	874 (53.8)	300 (57.5)	574 (52.0)	
Exercise, *n* (%)				
Rarely	612 (37.6)	202 (38.7)	410 (37.1)	1.42 (0.49)
Sometimes	824 (50.7)	266 (51.0)	558 (50.5)	
Daily	190 (11.7)	54 (10.3)	136 (12.3)	
Drinking, *n* (%)				
None	961 (59.1)	323 (61.9)	638 (57.8)	6.28 (0.04)
Less than two units/day	640 (39.4)	196 (37.5)	444 (40.2)	
Two or more units/day	25 (1.5)	3 (0.6)	22 (2.0)	
Smoking, *n* (%)				
None	1532 (94.2)	492 (94.3)	1,040 (94.2)	0.48 (0.92)
10 cigarettes/day	47 (2.9)	15 (2.9)	32 (2.9)	
20 cigarettes/day	46 (2.8)	15 (2.9)	31 (2.8)	
30 or more cigarettes/day	1 (0.1)	0	1 0.1)	
Mental illness treatment, *n* (%)				
None	1442 (88.7)	456 (87.4)	986 (89.3)	4.04 (0.13)
Follow-up	13 (0.8)	2 (0.4)	11 (1.0)	
Past history	171 (10.5)	64 (12.3)	107 (9.7)

### Study 2 results

**Fig. 2:**
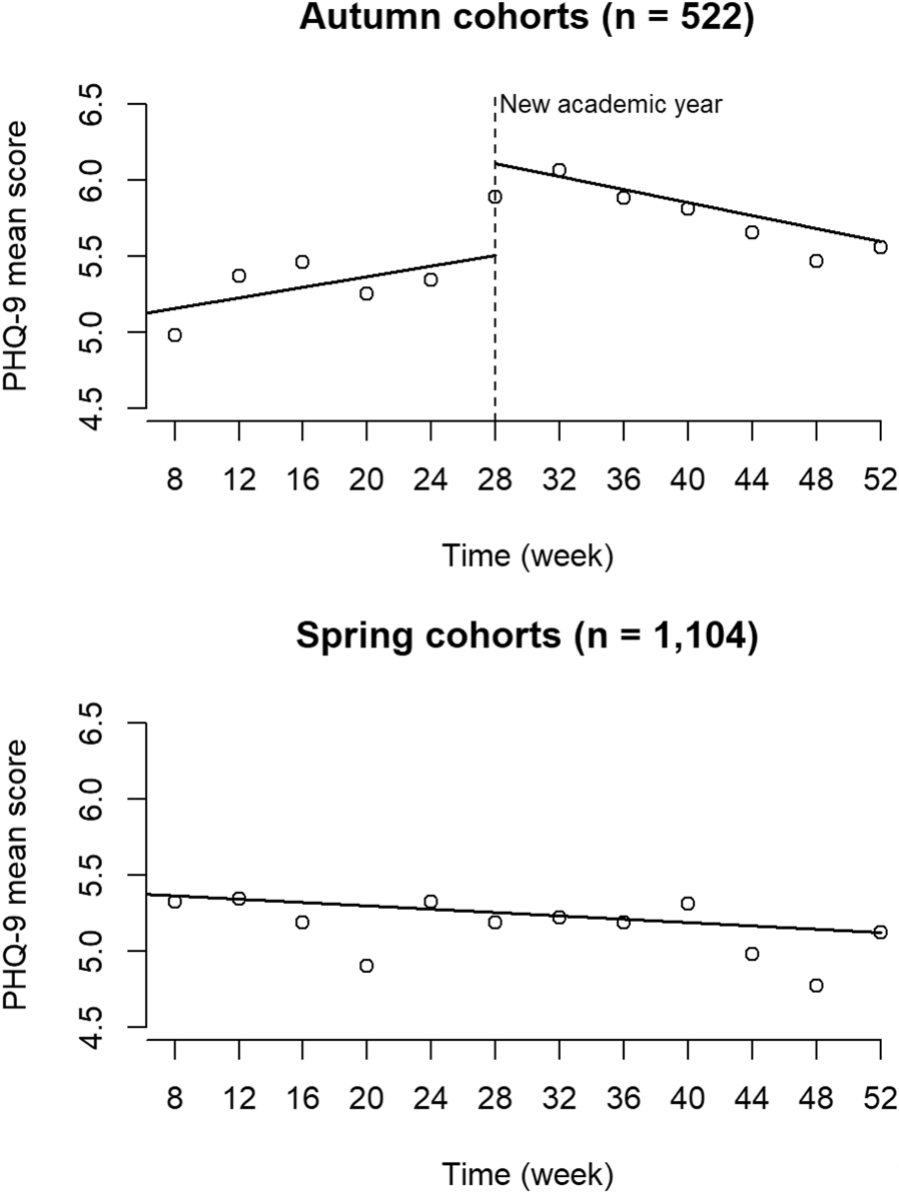
Interrupted and non-interrupted trajectories of depressive states among university students in the HCT’s seasonal cohorts. *HCT* Healthy Campus Trial, *PHQ-9* Patient Health Questionnaire-9

As shown in Table 1, participants in the autumn (*n* = 522) and spring cohorts (*n* = 1104) showed comparable sociodemographic characteristics. Except for the drinking category, there were no statistically significant variations in the distributions of most variables between the two seasonal cohorts. The two cohorts of the HCT illustrated distinctive trajectories in depressive states during the follow-up periods (Table 2 and Fig. 2). Our segmented regression analysis of the ITS data showed a significant level change of 0.60 points (95% CI 0.42–0.78; *p* < 0.001) at the 28-week evaluation point. PHQ-9 scores increased by 0.07 points every four weeks (95% CI 0.02–0.11; *p* = 0.12) following the intervention periods in the autumn cohorts. The upward slope changed by − 0.16 (95% CI − 0.21 to − 0.10; *p* = 0.003) immediately after the beginning of a new academic year. In contrast, PHQ-9 scores significantly decreased by 0.02 points every 4 weeks (95% CI − 0.03 to − 0.01; *p* = 0.008) through the follow-up periods in the spring cohorts.

**Table 2:** PHQ-9 scores of 1626 university students during the follow-up period in the HCT’s seasonal cohorts

Time	8w	12w	16w	20w	24w	28w	32w	36w	40w	44w	48w	52w
Autumn (*n* = 522)												
Valid %	93.3	74.1	73.6	68.8	77.6	62.3	60.9	56.1	56.1	55.9	54.8	81.4
PHQ–9												
Mean	5.0	5.4	5.5	5.3	5.3	5.9	6.1	5.9	5.8	5.7	5.5	5.6
Median	4.0	5.0	5.0	5.0	4.0	5.0	5.0	5.0	5.0	4.5	5.0	4.0
Range	0–22	0–22	0–21	0–20	0–27	0–25	0–27	0–23	0–24	0–20	0–23	0–26
IQR	2.0–7.0	2.0–7.0	2.0–8.0	2.0–8.0	2.0–8.0	2.5–8.0	2.0–9.0	2.0–9.0	2.0–8.0	2.0–9.0	2.0–8.0	2.0–8.0
Spring (*n* = 1104)												
Valid %	92.8	73.3	70.4	66.5	72.6	60.7	59.1	58.2	55.4	58.2	60.0	80.3
PHQ-9												
Mean	5.3	5.4	5.2	4.9	5.3	5.2	5.2	5.2	5.3	5.0	4.8	5.1
Median	4.0	4.0	4.0	4.0	4.0	4.0	4.0	4.0	4.0	4.0	4.0	4.0
Range	0–24	0–26	0–24	0–26	0–24	1–27	0–25	0–25	0–27	0–24	0–26	0–24
IQR	2.0–7.0	2.0–8.0	2.0–7.0	2.0–7.0	2.0–8.0	2.0–8.0	2.0–7.0	2.0–8.0	2.0–8.0	2.0–7.0	2.0–7.0	2.0–7.0

*HCT* Healthy Campus Trial, *IQR* interquartile range, *PHQ-9* Patient Health Questionnaire-9, valid % is the percentage of observations in each evaluation point out of the total number in the seasonal cohort

## Discussion

We investigated the effects of COVID-19 on depressive states across a sample of university students in Japan, focusing on the effects of four SOEs. Contrary to our first hypothesis, we found no evidence that the SOEs exacerbated depressive states. The first SOE appeared to be associated with a sudden increase in PHQ-9 scores during follow-up periods with the autumn cohort in 2019. However, this association was not confirmed for the other autumn and spring cohorts. Depressive states were commonly exacerbated at the 28-week evaluation points in all autumn cohorts, which corresponded to the beginning of a new academic year in April. To test our second hypothesis, we set a 28-week timeline for exposure to the new environment and thus identified a significant change in the level of depressive states.

Two previous studies reported worsened depressive symptoms among university students in Japan during the COVID-19 pandemic. One of these studies [31] compared the prevalence of PHQ-9 scores ≥ 10 among students at a national university between the first SOE (T1: May through June 2020) and the second and third SOEs (T2: March through May 2021). While the prevalence of moderate or severe symptoms increased from 11.5% at T1 to 16.6% at T2, only 36.3% of T1 participants (*n* = 2712) completed the T2 survey. In this regard, attrition bias likely influenced the increased prevalence, as participants who were not lost to follow-up may have been more conscious of their own health. In this study, the percentage of valid responses ranged between 36.4% and 98.2% during the follow-up periods in the autumn cohorts (Additional file 2), which might be subjected to attrition bias. However, the two seasonal cohorts with adequate evaluation points make it more plausible to investigate the impacts of the SOEs on depressive states in university students. The second study [23] compared annual data from health examinations among students at a national university from 2016 to 2020. The health assessments employed the PHQ-9 to screen for health concerns in approximately 12,600 students every April until 2019. As a COVID-19 countermeasure, screening was delayed until June in 2020; the 2020 mean score was 4.0, whereas those prior to 2020 ranged from 2.7 to 2.9. Based on the current study’s findings, we contend that the exacerbated symptoms in 2020 were caused by the timing of the evaluation points during exposure to the new academic year environment.

Psychological maladjustment can be an underlying mechanism in the significant exacerbation of depressive symptoms among university students during spring. This phenomenon is reflected by the term *gogatsubyou* (May blues), which gained use in the Japanese media during the 1960s. The term originally referred to apathetic states among new students who enrolled at universities after winning intense entrance examination races. Despite good performances on competitive exams, they tended to lack interest in academic life [32]. Gogatsubyou is considered to encompass the condition of student apathy proposed by Walters [33], in which college students prolonged the establishment of self-identity, as well as the moratorium that impeded them from facing reality. To date, the concept of *gogatsubyou* has expanded to include a state of temporary maladjustment in several contexts, such as advancing to the next grade, receiving an employment promotion, or entering higher education in April [34]. This culture-bound phenomenon is now regarded to be conceptually similar to adjustment disorders that are associated with depressed moods in the DSM-V [35].

### Limitations

This study has some limitations. First, the findings from Study 1 may be specific to Japanese political and social conditions. Indeed, previous meta-analyses found heterogeneous depression rates among different samples of university students during the COVID-19 pandemic [16–19]. In all such cases, international differences were contributing factors. The SOEs were implemented as a self-restraint policy in Japan, where semi-lockdown states may have imposed smaller effects on depression when compared to legally binding policies in other countries with unique epidemic situations. Second, the approach used in Study 2 did not account for exacerbating factors other than the new academic year environment. In this case, pollinosis may be important, as allergic rhinitis, which is estimated to affect 42.5% of individuals in Japan, especially in spring [36], is associated with both the seasonal elevation of inflammatory cytokines [37] and increased risk of depression [38, 39]. Third, the observational nature of this study could not ensure even distributions of unmeasured confounding factors between the two seasonal cohorts. Nevertheless, the ITS analysis allowed us to treat confounding factors within individual students as evenly distributed, both immediately before and after the new environmental exposure. Fourth, missing values in the PHQ-9 might depend on individual-level depression severity. Although mixed-effects models robustly handle missing values under the missing-at-random assumption, caution should be taken when interpreting our results owing to the potential for under- or over-estimations involving missing values.

## Conclusions

We found no evidence that the SOEs to control COVID-19 influenced changes in depressive states among Japanese university students who participated in the HCT follow-up periods. However, we did identify a sudden worsening of the conditions in new environmental exposure during the spring. This indicates that the onset of a new academic year may be a critical period for monitoring depressive states among university students, irrespective of the pandemic. Psychological maladjustment during this transition phase can be a significant contributor to exacerbated states. Hence, in clinical practice, there is a pressing need to provide targeted interventions and support during this period. During the pandemic, it is particularly important to monitor students with serious mental problems, including those at risk of committing suicide [40]. Nevertheless, our findings underline the necessity of robust mental health services in tackling issues that have continued to impact campus life before and since COVID-19.

### Supplementary Information

**Additional file 1:** Characteristics of 1626 university students in the individual cohorts of the Healthy Campus Trial.

**Additional file 2:** PHQ-9 scores of 1626 university students in the individual cohorts of the Healthy Campus Trial.

## Data Availability

The datasets analyzed in this study are available from the corresponding author upon request.

## Abbreviations

CBT: Cognitive-behavioral therapy
COVID-19: Coronavirus disease 2019
HCT: Healthy Campus Trial
ITS: Interrupted time series
PHQ-9: Patient Health Questionnaire-9
SOEs: States of emergency
