# Supplementary material for: Dynamics of depressive states among university students in Japan during the COVID-19 pandemic: an interrupted time series analysis
Source: Ann Gen Psychiatry. 2023 Oct 10;22:38. doi: 10.1186/s12991-023-00468-9 (PMC10563354; doi:10.1186/s12991-023-00468-9)
Supplement: Supplementary file 1 — Additional file 1: Characteristics of 1626 university students in the individual cohorts of the Healthy Campus Trial. [file 12991_2023_468_MOESM1_ESM.docx]

**Additional file 1.** Characteristics of 1,626 university students in the individual cohorts of the Healthy Campus Trial

| Characteristics | 2018 Autumn  (n = 55) | | 2019 Autumn  (n = 147) | | | 2020 Autumn  (n = 320) | | 2019 Spring  (n = 249) | | 2020 Spring  (n = 394) | | 2021 Spring  (n = 461) | |
| --- | --- | --- | --- | --- | --- | --- | --- | --- | --- | --- | --- | --- | --- |
| Age, mean (range), years | 23.0 | (19–30) | 21.7 | | (18–35) | 21.0 | (18–35) | 23.0 | (19–39) | 21.5 | (18–34) | 21 | (18–39) |
| Sex, n (%) |  |  |  | |  |  |  |  |  |  |  |  |  |
| Male | 29 | (52.7) | 78 | | (53.1) | 110 | (34.4) | 136 | (54.6) | 150 | (38.1) | 190 | (41.2) |
| Female | 26 | (47.3) | 69 | | (46.9) | 210 | (65.6) | 113 | (45.4) | 244 | (61.9) | 271 | (58.8) |
| Academic levels, n (%) |  |  |  | |  |  |  |  |  |  |  |  |  |
| Undergraduate | 35 | (63.6) | 104 | | (70.7) | 277 | (86.6) | 140 | (56.2) | 318 | (80.7) | 376 | (81.6) |
| Master | 17 | (30.9) | 31 | | (21.1) | 38 | (11.9) | 77 | (30.9) | 57 | (14.5) | 71 | (15.4) |
| Doctorate | 3 | (5.5) | 12 | | (8.2) | 5 | (1.6) | 32 | (12.9) | 19 | (4.8) | 14 | (3.0) |
| Living status, n (%) |  |  |  | |  |  |  |  |  |  |  |  |  |
| Living with family | 11 | (20.0) | 53 | | (36.1) | 140 | (43.8) | 52 | (20.9) | 191 | (48.5) | 177 | (38.4) |
| Living alone | 38 | (69.1) | 81 | | (55.1) | 165 | (51.6) | 174 | (69.9) | 184 | (46.7) | 260 | (56.4) |
| Living in a dormitory | 4 | (7.3) | 11 | | (7.5) | 12 | (3.8) | 17 | (6.8) | 9 | (2.3) | 17 | (3.7) |
| Others | 2 | (3.6) | 2 | | (1.4) | 3 | (.9) | 6 | (2.4) | 10 | (2.5) | 7 | (1.5) |
| Part-time employment, n (%) |  |  |  | |  |  |  |  |  |  |  |  |  |
| None | 10 | (18.2) | 21 | (14.3) | | 79 | (24.7) | 59 | (23.7) | 57 | (14.5) | 139 | (30.2) |
| A few times/year | 6 | (10.9) | 17 | (11.6) | | 31 | (9.7) | 31 | (12.4) | 37 | (9.4) | 26 | (5.6) |
| One to three times/month | 17 | (30.9) | 32 | (21.8) | | 53 | (16.6) | 62 | (24.9) | 93 | (23.6) | 73 | (15.8) |
| Two to three times/week | 17 | (30.9) | 64 | (43.5) | | 133 | (41.6) | 83 | (33.3) | 175 | (44.4) | 184 | (39.9) |
| More than four times/week | 5 | (9.1) | 13 | (8.8) | | 24 | (7.5) | 14 | (5.6) | 32 | (8.1) | 39 | (8.5) |

| Characteristics | | 2018 Autumn  (n = 55) | | | | 2019 Autumn  (n = 147) | | | 2020 Autumn  (n = 320) | | 2019 Spring  (n = 249) | | 2020 Spring  (n = 394) | | 2021 Spring  (n = 461) | |
| --- | --- | --- | --- | --- | --- | --- | --- | --- | --- | --- | --- | --- | --- | --- | --- | --- |
| Breakfast, *n* (%) |  | |  | |  | | |  |  |  |  |  |  |  |  |  |
| Rarely | 4 | | (7.3) | | 13 | | | (8.8) | 28 | (8.8) | 29 | (11.6) | 41 | (10.4) | 56 | (12.1) |
| Sometimes | 27 | | (49.1) | | 53 | | | (36.1) | 97 | (30.3) | 79 | (31.7) | 150 | (38.1) | 175 | (38.0) |
| Daily | 24 | | (43.6) | | 81 | | | (55.1) | 195 | (60.6) | 141 | (56.6) | 203 | (51.5) | 230 | (49.9) |
| Exercise, *n* (%) |  | |  | |  | | |  |  |  |  |  |  |  |  |  |
| Rarely | 24 | | (43.6) | | 63 | | | (42.9) | 115 | (35.9) | 92 | (36.9) | 145 | (36.8) | 173 | (37.5) |
| Sometimes | 27 | | (49.1) | | 71 | | | (48.3) | 168 | (52.5) | 130 | (52.2) | 195 | (49.5) | 233 | (50.5) |
| Daily | 4 | | (7.3) | | 13 | | | (8.8) | 37 | (11.6) | 27 | (10.8) | 54 | (13.7) | 55 | (11.9) |
| Drinking, *n* (%) | |  | |  | |  |  | |  |  |  |  |  |  |  |  |
| None | | 21 | | (38.2) | | 83 | (56.5) | | 219 | (68.4) | 99 | (39.8) | 214 | (54.3) | 325 | (70.5) |
| Less than two units/day | | 33 | | (60.0) | | 63 | (42.9) | | 100 | (31.3) | 141 | (56.6) | 174 | (44.2) | 129 | (28.0) |
| Two or more units/day | | 1 | | (1.8) | | 1 | (.7) | | 1 | (.3) | 9 | (3.6) | 6 | (1.5) | 7 | (1.5) |
| Smoking, *n* (%) | |  | |  | |  |  | |  |  |  |  |  |  |  |  |
| None | | 49 | | (89.1) | | 139 | (94.6) | | 304 | (95.0) | 231 | (92.8) | 374 | (94.9) | 435 | (94.4) |
| 10 cigarettes/day | | 4 | | (7.3) | | 3 | (2.0) | | 8 | (2.5) | 11 | (4.4) | 10 | (2.5) | 11 | (2.4) |
| 20 cigarettes/day | | 2 | | (3.6) | | 5 | (3.4) | | 8 | (2.5) | 6 | (2.4) | 10 | (2.5) | 15 | (3.3) |
| 30 or more cigarettes/day | | 0 | |  | | 0 |  | | 0 |  | 1 | (.4) |  |  |  |  |
| Mental illness treatment, *n* (%) | |  | |  | |  |  | |  |  |  |  |  |  |  |  |
| None | | 48 | | (87.3) | | 123 | (83.7) | | 285 | (89.1) | 219 | (88.0) | 354 | (89.8) | 413 | (89.6) |
| Follow-up | | 0 | |  | | 2 | (1.4) | | 0 |  | 3 | (1.2) | 2 | (.5) | 6 | (1.3) |
| Past history | | 7 | | (12.7) | | 22 | (15.0) | | 35 | (10.9) | 27 | (10.8) | 38 | (9.6) | 42 | (9.1) |
