# Supplementary material for: Dynamics of depressive states among university students in Japan during the COVID-19 pandemic: an interrupted time series analysis
Source: Ann Gen Psychiatry. 2023 Oct 10;22:38. doi: 10.1186/s12991-023-00468-9 (PMC10563354; doi:10.1186/s12991-023-00468-9)
Supplement: Supplementary file 2 — Additional file 2: PHQ-9 scores of 1626 university students in the individual cohorts of the Healthy Campus Trial. [file 12991_2023_468_MOESM2_ESM.docx]

**Additional file 2.** PHQ-9 scores of 1,626 university students in the individual cohorts of the Healthy Campus Trial

| **Time** | **0w** | **4w** | **8w** | **12w** | **16w** | **20w** | **24w** | **28w** | **32w** | **36w** | **40w** | **44w** | **48w** | **52w** |
| --- | --- | --- | --- | --- | --- | --- | --- | --- | --- | --- | --- | --- | --- | --- |
| 2018A (*n* = 55) |  |  |  |  |  |  |  |  |  |  |  |  |  |  |
| Valid % | 100 | 94.5 | 98.2 | 72.7 | 72.7 | 56.4 | 58.2 | 45.5 | 40.0 | 36.4 | 43.6 | 40.0 | 41.8 | 78.2 |
| PHQ-9 mean | 6.4 | 6.6 | 5.7 | 5.9 | 5.7 | 6.5 | 5.0 | 6.8 | 7.4 | 6.9 | 7.1 | 6.7 | 5.9 | 5.9 |
| PHQ-9 median | 6.0 | 5.0 | 5.0 | 5.0 | 4.5 | 6.0 | 5.0 | 6.0 | 5.0 | 5.5 | 5.5 | 5.5 | 5.0 | 5.0 |
| PHQ-9 range | 0–15 | 0–18 | 0–15 | 0–15 | 0–21 | 1–20 | 0–11 | 1–19 | 0–27 | 0–23 | 0–23 | 1–19 | 0–19 | 0–23 |
| 2019A (*n* = 147) |  |  |  |  |  |  |  |  |  |  |  |  |  |  |
| Valid % | 100 | 90.5 | 92.5 | 72.8 | 71.4 | 72.8 | 78.9 | 65.3 | 58.5 | 57.1 | 54.4 | 55.8 | 55.1 | 76.9 |
| PHQ-9 mean | 5.9 | 5.5 | 5.1 | 5.1 | 5.5 | 4.8 | 5.4 | 5.8 | 5.8 | 6.1 | 5.8 | 6.0 | 5.5 | 5.5 |
| PHQ-9 median | 5.0 | 5.0 | 4.0 | 4.0 | 5.0 | 4.0 | 4.5 | 5.0 | 5.0 | 6.0 | 5.0 | 5.0 | 5.0 | 4.0 |
| PHQ-9 range | 1–21 | 0–15 | 0–22 | 0–22 | 0–16 | 0–20 | 0–22 | 0–17 | 0–21 | 0–19 | 0–20 | 0–19 | 0–20 | 0–21 |
| 2020A (*n* = 320) |  |  |  |  |  |  |  |  |  |  |  |  |  |  |
| Valid % | 100 | 90.9 | 92.8 | 75.0 | 74.7 | 69.1 | 80.3 | 63.7 | 65.6 | 59.1 | 59.1 | 58.8 | 56.9 | 84.1 |
| PHQ-9 mean | 6.4 | 5.4 | 4.8 | 5.4 | 5.4 | 5.3 | 5.3 | 5.8 | 6.0 | 5.7 | 5.7 | 5.4 | 5.4 | 5.5 |
| PHQ-9 median | 6.0 | 5.0 | 4.0 | 5.0 | 5.0 | 5.0 | 4.0 | 5.0 | 5.0 | 5.0 | 5.0 | 4.0 | 4.5 | 4.0 |
| PHQ-9 range | 0–19 | 0–19 | 0–20 | 0–20 | 0–21 | 0–20 | 0–27 | 0–25 | 0–27 | 0–18 | 0–24 | 0–20 | 0–23 | 0–26 |
| 2019S *(n* = 249) |  |  |  |  |  |  |  |  |  |  |  |  |  |  |
| Valid % | 100 | 88.4 | 91.2 | 63.9 | 58.2 | 56.2 | 56.2 | 49.8 | 51.4 | 51.4 | 49.4 | 49.4 | 52.2 | 77.1 |
| PHQ-9 mean | 6.4 | 5.4 | 5.3 | 5.6 | 4.7 | 4.8 | 5.3 | 5.1 | 5.2 | 5.2 | 5.5 | 4.7 | 4.6 | 5.2 |
| PHQ-9 median | 6.0 | 5.0 | 4.0 | 4.0 | 3.0 | 4.0 | 4.0 | 4.0 | 4.0 | 4.0 | 5.0 | 3.0 | 4.0 | 4.0 |
| PHQ-9 range | 0–18 | 0–18 | 0–21 | 0–21 | 0–18 | 0–20 | 0–24 | 0–24 | 0–20 | 0–23 | 0–27 | 0–22 | 0–23 | 0–24 |

| **Time** | **0w** | **4w** | **8w** | **12w** | **16w** | **20w** | **24w** | **28w** | **32w** | **36w** | **40w** | **44w** | **48w** | **52w** |
| --- | --- | --- | --- | --- | --- | --- | --- | --- | --- | --- | --- | --- | --- | --- |
| 2020S (*n* = 394) |  |  |  |  |  |  |  |  |  |  |  |  |  |  |
| Valid % | 100 | 93.1 | 93.4 | 79.4 | 76.1 | 71.3 | 70.8 | 64.0 | 61.4 | 59.1 | 55.8 | 57.9 | 56.6 | 77.9 |
| PHQ-9 mean | 6.5 | 5.5 | 5.1 | 5.4 | 5.3 | 4.8 | 5.3 | 5.3 | 5.2 | 4.9 | 5.3 | 4.9 | 4.6 | 5.1 |
| PHQ-9 median | 6.0 | 5.0 | 4.0 | 4.0 | 4.0 | 4.0 | 4.0 | 4.0 | 4.0 | 4.0 | 4.0 | 4.0 | 4.0 | 4.0 |
| PHQ-9 range | 0–19 | 0–22 | 0–21 | 0–23 | 0–19 | 0–20 | 0–21 | 0–20 | 0–25 | 0–21 | 0–27 | 0–20 | 0–20 | 0–23 |
| 2021S (*n* = 461) |  |  |  |  |  |  |  |  |  |  |  |  |  |  |
| Valid % | 100 | 89.8 | 93.3 | 73.1 | 72.0 | 67.9 | 82.9 | 63.8 | 61.2 | 61.0 | 58.4 | 63.3 | 67.0 | 84.2 |
| PHQ-9 mean | 6.4 | 5.5 | 5.6 | 5.2 | 5.3 | 5.0 | 5.4 | 5.1 | 5.3 | 5.4 | 5.3 | 5.2 | 4.9 | 5.2 |
| PHQ-9 median | 6.0 | 4.0 | 4.0 | 4.0 | 4.0 | 4.0 | 4.0 | 4.0 | 4.0 | 4.0 | 4.0 | 4.0 | 4.0 | 4.0 |
| PHQ-9 range | 0–18 | 0–20 | 0–24 | 0–26 | 0–24 | 0–26 | 0–23 | 0–27 | 0–21 | 0–25 | 0–24 | 0–24 | 0–26 | 0–22 |

*Note:* PHQ-9, Patient Health Questionnaire-9; Valid % is the percentage of observations in each evaluation point out of the total number in the spring (S) cohort.
